# Second Annual Report of the Registrar-General of Births, Deaths, and Marriages in England. Presented to Both Houses of Parliament, by Command of Her Majesty

**Published:** 1841-04

**Authors:** 


### Art. VII.

*Second Annual Report of the Registrar-General of Births, Deaths, and Marriages in England. Presented to both Houses of Parliament, by command of her Majesty.—London,* 1840. Folio, pp. 165. 8vo, pp. 247.

We hail with satisfaction this Second Report from the Registrar-general. It comprises returns of births, deaths, and marriages, registered for the year commencing July 1, 1838, and terminating June 30, 1839, with copious illustrative tables. It is accompanied, as was the First Report, with an Appendix from Mr. Farr, which in the present instance contains remarks on the mortality and diseases of 1838, various tables on their relative prevalence in town and country, remarks on the influence of localities, pursuits, and the various circumstances comprised in the word hygiène on such prevalence, and valuable information on the progress of epidemics. We shall endeavour to present a condensed view of the contents of the Report and its Appendix.

The numbers registered in the year ending June 30, 1839, were births 410,540, deaths 331,007, and marriages 121,083, being an increase in the first and third classes, and a decrease in the second class, compared with the preceding year. The increase in the return of births is ascribed by the registrar-general, to a diffusion of the knowledge of the beneficial tendency of registration, whilst he considers that there was a deficiency in the marriages of the preceding year, in consequence of many, from a misapprehension of the object and effect of the recent act, having been solemnized immediately before the act came into operation.

The decrease in the reports of deaths is not supposed to be dependent on imperfect registration, or any adventitious circumstance, but really from there having been fewer to report, Mr. Lister, considering that the mortality during the preceding year was above the average, owing to the inclemency of the weather in the beginning of 1838, and to some epidemics which have subsequently declined in prevalence and severity.

We are informed that at present it is impossible to do more than approximate to a solution of the important question, what is the proportion of the mortality to the population of England and Wales? The following is his approximation: The population of the country, calculated from the census of 1821 and 1831, according to principles pointed out in the first report and explained in our review of that document, was, January 1, 1838, males 7,612,967, females 7,828,768, total 15,441,734; and January 1, 1839, males 7,723,924, females 7,942,876, total 15,666,800. The deaths registered during the same periods were, for the year ending June 30, 1838, males 170,965, females 164,991, total 335,956; for the year ending June 30, 1839, males 169,112, females 161,895, total 331,007. This shows the mortality to have been in 1837-8, one in 46; and in 1838-9, one in 47·3; and the mean of the two years to be one in 46·6; and, supposing 2 per 100 to be a sufficient correction for omissions in the registration, the mean mortality for these two years will have been one in 46.

Some changes are made in the arrangement of the abstracts in the present report as compared with the first. It will be remembered that in the first report the deaths at each successive year of life were exhibited; but in the present one the deaths during the first year are divided into six periods; during the four following years, they are shown for each separate year, and after that for quinquennial periods.

The reasons for the minute subdivision of the first year are the number of deaths which then took place, as was shown in the former report, where they were found to form more than a fifth of the whole mortality of the kingdom, and the rapid change in the expectation of life that then occurs. After the first year the ratio of mortality rapidly declines and this is shown by the enumeration of deaths for the four following years. The registers do not admit of a subdivision of these years, and did they do so, it would appear to us needlessly minute to follow them. By the adoption of the quinquennial period for the succeeding stages of life, a considerable source of inaccuracy is avoided, or at least its effect is diminished. The source to which allusion is made is the disposition "to speak in round numbers." This is strikingly exemplified in an abstract of ages, extracted from the burial registers of England and Wales, and published in the population abstract for 1831, in which table there is manifested, evidently from this habit, a striking increase of mortality at each decennial period from thirty to seventy inclusive, compared with the deaths in the intermediate years. Mr. Lister is of opinion that no enumeration, either of the living or the dead, more minute than for quinquennial periods can ever be made with success, and it must ever be remembered, that correct tables of mortality, require for their construction these two series of facts—the numbers living at different ages, and the numbers dying at the same ages, and the observed relation between those facts. He has adduced high authorities, British and foreign, for abandoning the attempt to enumerate accurately these two classes of facts year by year.

Mr. Farr, in his appendix to the report, bestows his attention first on the diseases of males and females. He estimates that the mortality of males was 7 per cent. higher than that of females, and remarks that it is well established that the mean duration of life is longer in females than in males. This discrepancy, it is obvious, may arise from dissimilarity of habits and occupations, difference of organization, or both conjointly. Mr. Farr considers justly that to refer the difference to the first set of causes exclusively, would be to take too narrow ground, for the differential mortality is greater in early childhood and before birth than in more advanced ages. This throws us for explanation on a difference in original structure. In what, however, this difference consists we are ignorant, and equally ignorant are we of the causes of the varying prevalence of certain diseases in the two sexes; as the reporter expresses it, we are not "aware that any anatomical or physiological reason has been advanced to explain the singular fact, that 620 males, and 1828 females died of cancer; 4026 males and 5071 females of hooping-cough; 27,935 males and 31,090 females of consumption; 4242 males and 3430 females of hydrocephalus; or, 152 males and 55 females of diabetes." It is no explanation certainly, but it may not be unworthy of remark, that in such of the diseases here mentioned as have a certain seat, the excess in the case of males is in affections of the urinary organs and of the nervous system, and in that of females in those of the organs of respiration: "34,321 males and 33,556 females died of the epidemic class of diseases; smallpox, measles, croup, thrush, diarrhœa, dysentery; cholera, and influenza proved most fatal to males; hooping-cough to females; typhus, scarlatina, and erysipelas, were scarcely more fatal to males than to females." There was a small decrease in this (the epidemic) class, as compared with 1837, viz. from 4·7 per 1000 to 4·5 per 1000 of the living. The 67,877 deaths from this class compose 21 per cent. of the total specified deaths, whilst in 1837, the proportion was 23 per cent. Of the 4·5 deaths to the 1000 of the living, 1·0 arose from smallpox and 1·3 from typhus. We are convinced that the former item will surprise and grieve many; but this is a subject to which we shall have subsequent occasion to revert.

Diseases of the nervous system destroyed 49,704 persons, or produced about 14 per cent. of the total deaths. Of these deaths 26,047 are classed under the vague head, convulsions; 7612, under that of hydrocephalus; 2178 are attributed to cephalitis. Regarding hydrocephalus, Mr. Farr remarks that, like consumption or mesenteric disease, it is a modification of scrofula, in which view we concur with him, notwithstanding the recently promulgated contrary opinions of Dr. Davis. Mr. Farr justly remarks that this disease is very fatal in ill-ventilated dirty neighbourhoods, where the inhabitants are poor and the mothers ill-fed. Are these the localities in which acute phlogoses arise? Are they not rather the haunts of scrofula? When we have seen hydrocephalus in the abodes of the wealthy, we have ever had reasons (independent of this disease) for considering the families scrofulous.

Diseases of the nervous system are 23 per cent. more fatal to males than females, the difference being chiefly in infantile diseases. Apoplexy destroyed more males than females—paralysis more females than males. To chorea (St. Vitus's dance) the deaths of four males and twenty females were ascribed—a smaller number than we should have expected, as we have repeatedly seen this disease fatal, and more frequently so to females than males. To delirium tremens were ascribed the deaths of 167 males and 15 females. We should think from our own observation this statement also, especially as it regards males, considerably below the truth; and there is an obvious motive for inaccuracy, for the term delirium tremens is now familiar among all classes, and by no means in good repute. Deaths took place from tetanus in 100 males and 29 females; but it is justly remarked that men are more exposed in a similar proportion to the injuries from which it generally originates. The conclusion of the whole is that, however certain diseases of this class may have proved more fatal to females than males, the latter suffer more from diseases of the nervous system than the former, in the proportion of 23 per 100.

Diseases of the respiratory organs produced 90,823 deaths, that is, a mortality of 6·0 per 1000; while the annual mortality of the group in 1837 was 5·5 in 1000, or 11 per cent. less. The following is Mr. Farr's account of the variations in this class of diseases, occasioning the difference in favour of the last year. The mortality of consumption fell from 3·96 to 3·93 in 1000; that of pneumonia, bronchitis, and pleurisy, rose from 0·93 to 1·38 (69 per cent.); asthma from 2·5 to 3·8 (52 per cent.); 3·8 in 1000 males and 4·1 in 1000 females died of *consumption;* 11,691 males and 9488 females died of inflammatory affections of the throat, larynx, air-tubes, lungs, and pleura. He adds that consumption is 8 per cent. more fatal to females than to males. He specifies some errors in the registers for which allowance must be made, such as many cases (1218) referred to hemorrhage and registered ruptured blood-vessel, which he considers to belong to consumption. In this view we fully concur with him, having in every instance which we have had an opportunity of examining found the disease thus designated to consist of hemorrhage into a tubercular cavity. With these qualifications, 27½ per cent. of the total deaths were due to diseases of the respiratory system, and 18 per cent. to consumptions; namely, 16·0 per cent. of the deaths of males and 19·2 of the deaths of females.

These statements correspond very exactly with those of Mr. Farr for the former year, and which we transferred to our review of his Letter. In that document as in this there was shown an excess in the inflammatory class of diseases of the respiratory organs in males, but an excess of phthisis in the other sex brought the deaths from the whole class to an equality in the two. In the present Report we find the general equality not perfect, there being a slight excess in the deaths of females on the total mortality of the class, the proportion being 271 males to 278 females, owing to the preponderance of phthisis in the latter sex. On this subject Mr. Farr makes the following very important remarks:

"The higher mortality of English women by consumption may be ascribed partly to the in-door life which they lead, and partly to the compression preventing the expansion of the chest by costume. In both ways they are deprived of free draughts of vital air, and the altered blood deposits tubercular matter with a fatal, unnatural facility. 31,090 English women died in one year of the incurable malady! Will not this impressive fact induce persons of rank and influence to set their countrywomen right in the article of dress, and lead them to abandon a practice which disfigures the body, strangles the chest, produces nervous or other disorders, and has an unquestionable tendency to implant an incurable hectic malady in the frame? Girls have no more need of artificial bones and bandages than boys." (Letter to the Registrar-General, p. 73.)

We entirely concur in the reprobation of this unhealthy and disfiguring practice; but the other faulty arrangement pointed out by Mr. Farr is equally deserving of censure. The daughters of the poorer class are "deprived of free draughts of vital air," and, what we think equally important, of that free exercise, without which the circulation is languid and the blood vitiated, when as dress-makers they are confined all day and a portion of the night in crowded and noisome appartments, ministering to the vanity and, by the tight garments they fashion, to the deterioration of the health of the upper class. Of the daughters of this latter class the health is sacrificed to those eternal *accomplishments*, which, with small benefit to the intellect and none to the feelings, inevitably dwarf and dwindle the body, and too often lay the beauteous fabric in the dust. Many hours to music, many to drawing, many to fancy-work, some to languages, and few—but very few—to exercise in the open air "wear through the longest day." When exercise is taken it is often in some public walk, under the superintendence of some silly and ignorant governess, by whom every ebullition natural and healthful to youth—the jocund laugh, the run and the leap—are repressed as ungenteel—that stupid and vulgar word to which so much of the health and happiness of youth is sacrificed. In an education calculated to draw forth the powers of the mind and body all should be vigorously done. But this is overlooked in female education. During the long hours of lessons in drawing, music, &c., the attention becomes languid, the mind weary, objects impress it feebly, and much of the time that is thus literally wasted would be infinitely much more usefully spent in play. A shorter period of vigorous study and a longer one of bodily exertion of a different kind from what we have witnessed and endeavoured to describe, would send young ladies forth to the world from their homes or seminaries of education at once better instructed and more healthful than we now see them.

1·205 per cent. of the total deaths in males, and ·945 per cent. of those in females, or 2032 of the former, and 1530 of the latter, are registered as having died of diseases of the heart and blood-vessels. This Mr. Farr remarks, and with justice, is below the true number; and he ascribes the deficiency to the art of auscultation not being sufficiently diffused. Much may be justly ascribed to this; but something, too, may be attributed, in the numerous cases in which death occurs suddenly, to the mode in which inquests are conducted. The coroner and the jury learn enough to show that the death has not been unnatural or violent: with this they are satisfied, and the customary but unmeaning verdict, "Died by the visitation of God," is returned: having no evidence of the actual pathological cause of death, they do not direct the only means of revealing it—an anatomical examination. Aneurism destroyed three times as many males (88) as females (31), the same proportion as was observed in 1837. Diseases of the digestive organs were less fatal than in the latter end of 1837, the mortality having declined from 1·4 to 1·3

per 1000, or, including thrush, diarrhœa, dysentery, and cholera, from 2·07 to 1·59 per 1000. The proportions in the two sexes were 5·966 per cent. males, and 5·709 per cent. females of the total deaths. There was an increase in the deaths from teething. Stricture of the intestines was, as might be supposed from its being very generally of a cancerous nature, more frequent in females than males. Of hernia there died 318 males and 189 females; of peritonitis, 51 males and 117 females, a proportion of the latter cases probably puerperal. Inclusive of teething, 10,992 persons died of diseases of the stomach and bowels; 3 of diseases of the pancreas; 3880 of diseases of the liver (including jaundice, 841); and 27 of diseases of the spleen. The 1385 classed under "disease of the intestinal canal," comprised cases of chronic enteritis, gastritis, dyspepsia, as well as some malignant diseases.

"1338 males and 313 females died of diseases of the urinary organs. The mortality of the former from stone and gravel was 4 in 100,000; of the latter, 0·5. The difference in the seven heads is exaggerated by, but cannot be exclusively attributed to, mechanical causes." (Letter to the Registrar-General, p. 73.)

The heads mentioned in this Report are, nephritis, ischuria, diabetes, cystitis, stone, stricture, and disease. Now under every one of these heads we find more cases in the male than the female subject, and some of the diseases appear to owe no aggravation to mechanical causes. We find, for instance, under that of nephritis 113 cases occurred in males, and only 44 in fèmales; of diabetes 152 in males, and 55 in females; of cystitis 103 in males, and 25 in females; and under the general term disease 578 in males, and 132 in females; so that there can be little doubt of the greater tendency to diseases of the urinary organs in males quite independent of any mechanical cause. So far indeed as these very valuable reports have proceeded they have manifested a greater tendency to diseases of the nervous system and the urinary organs in the male, and of the organs of respiration in the female sex; whilst among diseases of uncertain seat, they have shown a very decided predominance of cancer in females.

The diseases of *uncertain seat* proved fatal to 21,871 males and 22,361 females. Of this class dropsy was observed in 5170 males and 7172 females; hemorrhage in 730 males, 488 females; mortification in 802 males and 541 females; malformation in 93 males and 73 females; scrofula in 599 males, and 520 females; and carcinoma in 620 males and 1828 females. Purpura, formerly referred to diseases of the skin, where it was manifestly misplaced, is now very appropriately transferred to this class. Of this singular disease there died 31 males and 27 females. Mr. Farr remarks respecting it that it appears to consist in an alteration of blood, in which view he is probably correct. But we question whether the alteration be of one kind in all cases, having certainly seen the disease in two different if not opposite conditions, the one having relation to active hemorrhage and requiring depletion for its cure, the other being of the nature of passive hemorrhage and requiring an opposite management. The deaths from hemorrhage were 730 males, 488 females, being in the proportion of 1·5 of the former to one of the latter; but we have already coincided in a suspicion expressed by Mr. Farr, that many of these cases belong to consumption. The united deaths from all forms of dropsy (hydrocephalus included, which belongs to this disease only in name) amount to, males 10,734, females 11,694—total 22,428, showing the same predominance of dropsy in the female sex which was shown in last year's report. Of sudden deaths 1840 occurred in males, and 1172 in females, being in the proportion (including violent deaths, which are generally sudden) of 10 females to 18 males, the former having so much less chance of sudden death: 12 per cent. of the deaths of females, and 10 per cent. of the deaths of males were ascribed to old age and to natural decay.

The deaths from manifest external causes are arranged under three heads, viz. intemperance, starvation, and violent deaths. To the first head were ascribed the deaths of 195 males and 36 females. From starvation by cold or want there arose the deaths of 126 males and 41 females; and the violent deaths were 8359 males and 3368 females. These may again be divided into voluntary and involuntary. The numbers ascertained of the former class, suicides, were, males 751, females 307—total 1058, and in many cases of individuals found dead the agent was not ascertained. Relative to suicides we are furnished with two tables, which are of interest. According to the one the tendency to this crime increases till the age of 60, the rate of increase from 30 to 60 being 49·6 per cent. every 10 years. From the other table we learn that the tendency to suicide is the highest in the metropolis, and the least in Wales, the deaths in the former locality from this cause being in the ratio of 12·6 per 100,000 inhabitants, and but 2·5 in the latter, whilst the average ratio for England and Wales is 6·8 per 100,000. From a third table we learn that the smallest number of suicides occur in the cold season of the year.

The general summary of the causes of death given by Mr. Farr is, that 36,799 died from inflammations; 85,506 from specific inflammations; 19,122 from the terminations of inflammation; 15,125 from hemorrhages; 2821 from carcinomatous diseases; 60,868 from tuberculous diseases; 2256 from disordered secretions; 2512 from depraved nutrition; 44,773 from disorders of the nervous system; 35,564 from old age; and 11,727 from violent deaths.

The augmented fatality in an urban compared with a rural population received, it may be remembered, considerable attention in the report of last year, and the subject is continued in the present one. The following is Mr. Farr's estimate on a large scale of the relative mortality of city and country—an estimate formed on the same principle as that of table E in the report of last year. In table C then of the present report we are presented with an abstract of the deaths from twelve classes of diseases in city and country districts, the former with a population of 3,726,221, the latter with one of 3,539,908. In the former there perished from epidemic, endemic, and contagious diseases 23,655, in the latter 13,685; from diseases of the nervous system the numbers stood respectively 15,651, and 8177; from those of the respiratory organs 28,973 and 18,508; from those of the organs of circulation 1301 and 712; of the digestive organs 6505 and 3361; of the urinary organs 417 and 373; of the organs of generation 984 and 547; of the organs of locomotion 653 and 354; of the integumentary system 144 and 66; of uncertain seat 10,447 and 10,529; from age 7374 and 8874; violent deaths 3104 and 2516; and from causes not specified 1811 and 2708: forming a total of deaths in the cities of 101,019, and in the country of 70,410.

The annual rate of mortality in the cities was 2·7, in the counties 2·0 per cent.; and the mortality in the cities 1·36 to 1·00 in the counties. The mean duration of life in the two sets of circumstances would differ nearly in the ratio of 37 years and 50 years.

"In examining the special causes of death," says Mr. Farr, "three classes may be distinguished: one class which was exaggerated in cities to the highest pitch, a third class in which the mortality was nearly the same, or in excess in the counties, and an intermediate class. To 1·00 death in the counties, the deaths out of the same amount in the cities, were by asthma, 3·80; erysipelas, 2·71; convulsions and teething, 2·57; cephalitis and hydrocephalus, 2·41: hydrophobia, 2·37; pneumonia, bronchitis, and pleurisy, 1·99; delirium tremens, 1·98; typhus, 1·88; smallpox, 1·73; heart-disease, 1·73; child-birth, 1·63; syphilis, 1·59; rheumatism, 1·58; gout, 1·55; hernia, 1·48; purpura, 1·46; sudden deaths, 1·45; liver disease, 1·45; hepatitis, 1·35; tetanus, 1·32. The excess of mortality in cities, was less in the following cases: by consumption, 1·24; croup, 1·23; violent deaths, 1·17; stone, 1·11; mortification, 1·10; malformations, 1·07; apoplexy, 1·07; hemorrhage, 1·02. The mortality by the third class of causes, was greater in the counties than in the cities; for the mortality to 1·00 in the counties was in the cities, by paralysis, ·99; dropsy, ·99; jaundice, ·99; diabetes, ·97; cancer, ·92; hydrothorax, ·88; hematemesis, ·79; debility (frequently premature birth), ·75; atrophy, ·75; scrofula, ·46." (Letter to the Registrar-General, p. 81.)

In reference to these comparative numbers, whether in general or in particular, it should be borne in mind that the argument is stated in a form by no means favorable to a rural population, for in the districts comprising the population of 3,539,908, and furnishing the mortality of 70,410, several cities are included. The excess of mortality then in cities as now constructed, compared with districts strictly rural, is greater than is shown by these returns. Mr. Farr asks the very reasonable question; "is the excessive mortality of cities inevitable?" In our review of the first report, we ventured in a similar spirit to remark that the facts relative to the mortality of rural districts and towns "were deduced from a comparison between rural districts and English towns, the growth of a progressive civilization, and that the older parts of them, now chiefly inhabited by the labouring poor, were formed in the early stages of civilization. We should like much to see a comparison between the mortality of country districts, and large towns brought rapidly into existence, under a civilization fully developed—those of the United States for instance." Mr. Farr answers his own question by remarking that the aggregation of mankind in towns is not inevitably disastrous: and that health and life may be preserved in a dense population, provided the density be not carried beyond certain limits. He produces abundant evidence from metropolitan districts of the truth of the first of these propositions. With regard to the second we would remark, besides that "*certain* limits" like other certain things, seem to us the most *uncertain* possible, that nothing has impressed us more on examining the very valuable tables descriptive of the mortality in the metropolitan districts, than the want of connexion they display between density of population and shortness of life. On referring to table G, one of singular value, we find, for instance, that the metropolitan district of Bermondsey, with a space of 88 square yards to one person, has an annual mortality of 3·070 per cent.: Shoreditch, with 35 yards to one person, one of 2·982 per cent.; Westminster, with 72 yards to one person, a mortality of 3·424 per cent.; the Strand, with one exception, the densest population quoted, being but 18 square yards to one person, one of 2·535 per cent., that is a smaller mortality than Stepney, with 144 yards to one person; St. Martin's in the Fields, with a space of 52 yards to one person, has a mortality of 2·998 per cent.; and the city of London, with a space much smaller than this last, viz. 30 square yards to one person, has a mortality of 2·062 per cent. or the smallest of all the metropolitan districts, that of Hackney excepted. This last district is singularly contrasted with Camberwell. Hackney with a space 434 square yards to one person, has a mortality of 1·782 per cent.; Camberwell, with one of 557 yards to one person, has a mortality of 2·181 per cent.

Mr. Farr throws out suggestions for the improvement of the health of the metropolis. The primary objects to be kept in view are, the careful exclusion of all unnecessary animal and vegetable matter, the immediate removal of residual products, and the dilution of inevitable exhalations. He discusses the mode of accomplishing these objects by forbidding the burial of the dead among dwellings crowded with the living; the exclusion of unwholesome manufactories and of slaughter houses from densely peopled districts, and the improvement and extension of sewers. On this last subject he says, "if a survey were made of the districts of the metropolis, and the levels, the sewers, the drains, and the nuisances known to be pernicious, were accurately laid down upon a map, it would agree very remarkably with the table of relative mortality; and the construction of such a map would complete the view of the evil in all its details, and form the basis of a well-planned remedy." We believe in every city the sewerage and drainage to be among the most important elements—probably the most important element of health and longevity; and we think it probable that a map, such as Mr. Farr has mentioned, would clear up the apparent anomalies, on which we have remarked, in the population and mortality tables of the metropolitan districts.

The very difficult subject of climate does not escape the observation of the reporter. We are presented with tables containing in all probability valuable information on the subject, but information requiring to be disentangled from the variety of other circumstances, with which it is complicated and unavoidably confounded in its effects—such, for instance, as density of population, variety of occupation, and differences of food. We at this moment refer to such a table, showing the mean annual mortality, per cent., in the metropolis and different districts of England and Wales, by twelve classes of diseases. The districts seem well devised for showing various influences, and among others the influence of climate. They are, the Metropolis, Cheshire and Lancashire, Durham, Northumberland, Cumberland and Westmoreland, Gloucester, Herefordshire, Shropshire, Worcestershire, Staffordshire, and Warwickshire, Middlesex (part of), Hertfordshire, Bucks, Oxon, Northampton, Huntingdon, Bedford and Cambridge, Monmouth and Wales, Yorkshire, Surrey (part of), Kent (part of), Sussex, Hampshire and Berkshire, Wiltshire, Dorsetshire, Devonshire, Cornwall and Somersetshire, Essex, Suffolk and Norfolk, Leicestershire, Rutlandshire, Lincolnshire, Nottinghamshire and Derbyshire. To our eye, however, this very valuable table appears to have a stronger bearing on density of population, than on climate.

This density appears to manifest its effects chiefly in the production of three classes of diseases, the epidemic class, affections of the respiratory organs, and of the nervous system. The metropolis has a population of 26,903 persons to one square mile, whilst the average of England and Wales, is 269 per square mile. The metropolis loses ·742 per cent. by epidemic, endemic, and contagious diseases, and 219 per cent. by typhus, whilst the average deaths throughout the country are by the entire class ·452 per cent. and by typhus, ·125. Cheshire and Lancashire, with a population of·701 per square mile, mnch below the metropolis indeed, yet next to that the most densely-peopled district in England, loses by the whole epidemic class, ·490 per cent., and by typhus, ·150. Thus it seems that the most densely-populated districts have suffered most by the epidemic class; London having no rival in this respect, and Cheshire and Lancashire exceeding all the more thinly-peopled districts, with the exception of Monmouth and Wales, which district with a population of 126 per square mile loses ·630 per cent. by the whole epidemic class, and by smallpox, ·202 per cent., scarlatina, ·123 per cent., and by typhus, ·152 per cent. It is evidently true, as remarked by Mr. Farr, that the ordinary laws of mortality are at present disturbed in Wales, by the influx of workmen within the mining districts. The population suddenly collected is exposed to all the evils of dense districts, without the alleviations which spring up in towns of slow growth. The overt Chartist excesses, and the more lurking, but still close political organization which we know, pervades the district, indicate its unhealthy social condition, and, at the same time, aggravate the source whene they spring. Exclusive, however, of the anomaly presented by Wales, the increase of mortality in a dense population from the epidemic class of diseases, is manifested in this table.

This pernicious influence of a dense population on diseases of the respiratory organs appears to be unequivocally evinced by the same table. The average deaths throughout the country, from all diseases of the respiratory organs, are ·605 per cent. and from phthisis ·393 per cent., but in London, the deaths from diseases of the respiratory organs generally, are ·770 per cent., and from phthisis, ·414 per cent., and in Cheshire and Lancashire, they are from the former ·783 per cent., and from the latter, ·509 per cent., without there being an excess above the average in any other district worthy of mention. Again, diseases of the nervous system produce an annual average mortality of ·332 per cent., and the only excess beyond this is in the districts already so often referred to, the metropolis presenting a mortality from this source, of ·437 per cent., Cheshire and Lancashire, one of ·461 per cent.

Considering the mortality from these three sources in these the most densely-peopled districts in this country, we are disposed to attribute a good deal to density of population, especially in the epidemic class of disease; but the excess in the manufacturing districts is so great in diseases of the nervous and respiratory systems, especially in the latter, that when we consider, moreover, the relative population of the two districts, we are forced to acknowledge that some other instrument of destruction besides density of population is in action, and we naturally look to the close and foul air of cotton mills.

In some remarks on the influence of the seasons, supported, as his remarks always are, by reference to tables, the reporter shows that the well-known statement of Celsus on the same subject, "Saluberrimum ver est, proxime deinde ab hoc hiems, periculosior æstas, autumnus longé periculosissimus," is inapplicable to *our* seasons at present. The following return of deaths in the metropolis expresses, we believe, very correctly, the order of insalubrity throughout England. There were registered in the winter quarter of 1838, 15,611 deaths; in spring, 13,109; in summer, 11,397; and in autumn, 12,581. This order Mr. Farr considers to have prevailed in England ever since the beginning of the last century. Prior to this period, however, the order of insalubrity was the same in this country as that indicated by Celsus; the autumn, at least, was "longé periculosissimus." We believe the explanation of the extreme insalubrity of autumn in this country formerly, and in many parts of the world at present, to be, that it is the season when intermittent fever, and especially those forms of it to which we give the name of remittent, but which elsewhere are called pernicious, pestilential, and malignant intermittents, are most prevalent. The febres algidæ of Torti, and some of the worst forms of those described by Morton—diseases bearing, in many respects, no slight resemblance to Indian cholera—committed their ravages principally in autumn. Under the influence of civilization and cultivation, countries become much more healthy; but the first and greatest amelioration is manifested in the extinction of diseases originating in malaria, which was most widely diffused and malignant in autumn, and still is so where it exists, as many parts of Europe and some of our own colonies can testify. England itself, however, may be considered as civilized and cultivated beyond the malarious point, and hence our autumns have ceased to be the insalubrious seasons of the year, the preeminence in this respect being conceded to our rigorous winters.

Many of the causes of disease, remarks Mr. Farr, act with equal force from year to year; others regulated by the seasons, increase or decrease with the temperature. Epidemics, however, follow laws of their own, and, in this class, the greatest variety may be expected in these annual reports: they have been a fertile source of discussion. How far registration may throw light on their origin is doubtful, but it seems well suited to illustrate, at least, the laws of their propagation.

The smallpox epidemic is investigated at considerable length in the report throughout its prevalence, from July 1st, 1837, to December 31st, 1839; a period comprising ten quarters, and two winters, two springs, three summers, and three autumns. It was composed of a succession of smaller epidemics, and whether the commencement or the acmé be considered, it is evident that it was not influenced by such circumstances as temperature or change of seasons; for, at the time it was beginning in one district, it was at its height or was declining in another place apparently in the same circumstances. This is manifested by Mr. Farr's tables, and especially by tables (*m*) and (*n*), which will be found at page 92 of his letter.

The total deaths from the epidemic were 30,879, which occurred in the following numbers in the successive quarters: in the first quarter, comprising the months of July, August, and September, 1837, they were, 2543; in the second, 3289; in the third, 4242; in the fourth, 4489; in the fifth, 3685; in the sixth, 3851; in the seventh, 2982; in the eighth, 2505; in the ninth, 1533; and in the tenth, 1730: total, 30,789. The annual rate of mortality from the disease was 0·8 in 1000; in the metropolis it was 1·1, and in Monmouthshire and Wales 1·2 per 1000. From a careful survey of the very ample table (Table P, pp. 174-195), the epidemic appears to have visited the whole of England and Wales, but by no means occupying successive points: there are no indications of its migrating from district to district on a tour through the country. We find it throughout the whole of the ten quarters in the metropolis on the south-east, and in Sunderland, Tynemouth, Newcastle, and Berwick-on-Tweed in the north-east. It is observed, too, during the same period in Bath and and in Wales on the south-west, and in Carlisle and various other places in Cumberland on the north-west, whilst almost every supposable intermediate point is similarly affected. Mr. Farr appears to express the view which we certainly take, that these tables furnish no reasonable grounds for supposing that this epidemic was communicated from place to place. Adopting the illustration furnished by Liverpool and Manchester, between which two towns the intercourse is perhaps more intimate than between any towns in Europe, he remarks that isolated cases of smallpox existed all the while in Manchester; the seeds of the epidemic were there, and would not the causes which generated the epidemic in Liverpool, the place first attacked, have led to the same result in Manchester? "At any rate, the evolution of the epidemic in Liverpool could not be traced to external contagion, and the problem remains for solution—Why did the deaths from smallpox rise so rapidly, that at last 418 individuals perished in three months, while the ordinary mortality in Liverpool and West Derby from smallpox is 27 in three months?"

This vexed question the reporter very judiciously leaves undisturbed by further vexation, and confines himself to tracing the law of the diffusion of the epidemic; it increased up to the fourth of the ten periods into which the epidemic is divided, and the rate of increase was 30 per cent., or 1·30. This is shown in the first three successive numbers, 2513, 3829, and 4242. From the third to the fourth number (4489), the increase is only six per cent., or 1·06; it then remains stationary, as Mr. Farr describes it, like a projectile at the summit of the curve which it is destined to describe.

To calculate the rate of decrease, Mr. Farr takes the mean of the third and fourth numbers (namely 4365), and, calculating from this, finds the decrease to have proceeded at an accelerated rate, the rate of acceleration being 1·406, and the rates of decrease standing thus, 1·052, 1·101, 1·152, 1·205, 1·268, and 1·138; or, in other terms, 5, 10, 15, 20, 26, and 32 per cent.: the last seven numbers divided each by the corresponding number marking the rate of decrease, presents in the number next below, within an insignificant fraction, the quotient which should result arithmetically from such a division.

Finding it exceedingly interesting to be presented with definite proportions, where all has hitherto been vague, we shall proceed to show Mr. Farr's calculations of the same epidemic in the metropolis, where the mortality was greater than in all other parts of England. The deaths in London in the ten successive periods were, 257, 506, 753, 1145, 1061, 858, 364, 117, 65, 60. The rate of increase in the first and second periods was 1·97, and in the second, third, and fourth periods, it was 1·50. —The rate of decrease is thus stated by Mr. Farr. The mean registered quarterly deaths from the fourth period were 1103, 959, 611, 240, and 91. The calculated series was 1103, 967, 611, 278, 91 ; certainly a remarkable approximation. The number 1103 was the mean of the deaths registered in the fourth and fifth, 959 was the mean of those registered in the fifth and sixth periods, and the other numbers were obtained in the same manner. The first rate (the rate of decrease) of the calculated series was 1·14, and the other rates were obtained by multiplying 1·14 four times in succession by 1·39, the constant, or the rate of acceleration.

"The rates," it is added, "vary with the density of the population, the numbers susceptible of attack, the mortality. and accidental circumstances ; so that to obtain the mean rates applicable to the whole population, or to any portion of the population, several epidemics should be investigated. It appears probable, however, that the smallpox increases at an accelerated and then a retarded rate; that it declines first at a slightly accelerated, then at a rapidly accelerated, and lastly at a retarded rate, until the disease attains the *minimum* intensity and remains stationary." (Letter to the Registrar-general, p. 97.)

Tables are furnished, presenting a view of the progress of four other epidemics, measles, typhus, hooping-cough, and scarlatina in the metropolis, and an abstract of their contents will be found in table (*q*) in the modest form of a foot-note at the close of Mr. Farr's letter. He remarks that they exhibit the same regularity as smallpox, but considers that the laws which govern their course will be more conveniently discussed when the abstract of the observations has been extended over another year. There perished during the latter half of 1837 and the year 1838 in the metropolis, by measles 1942, by typhus 6011, by hooping-cough 3149, and by scarlatina 1942.

Mr. Farr has laboured this branch of his subject with great effect: it is one to which the present report owes much of its interest, and to which future reports will probably be equally indebted ; for whilst sporadic diseases hold nearly the same course annually, and must consequently elicit, if any, similar remarks, it is in the epidemic class that the greatest variety may be expected ; and the evolution of the laws of their diffusion will, in all probability, furnish forth the labours of many years. The interest which the reporter feels in this subject has led him to comment favorably on the hypothesis, which, though not originating with either of these writers, has received from Drs. Holland and Henle* much illustration and support—that the material of contagion is composed of organic matter. Supported as this doctrine is by much plausible analogy, we consider, notwithstanding, that we have designated it properly when we have called it an hypothesis, and regarded merely as such we do not object to it, but, on the contrary, highly approve of it, thinking well-devised hypotheses the best stepping-stones to substantial theories—believing the observation required for their comparison with actual phenomena to be the best means of bringing out the whole truth.

We have concluded our analysis of the medical part of this valuable volume, and should have concluded altogether, had not one point of extra-professional information incidentally, as it were, gathered from the

* See British and Foreign Medical Review, No. XVIII., p. 398.

reports of marriages, struck us as being one of so much interest, that we are disposed to communicate it to our readers ; it is, in fact, the statistics of education among all ranks of the adult population throughout the country, deduced in a mode which we shall leave Mr. Lister to explain in his own language.

"Almost every marriage is duly registered, and every register of marriage is signed by the parties married, those who are able writing their names, and those who are unable, or who write very imperfectly, making their marks; therefore an enumeration of the instances in which the mark has been made will show the proportion among those married who either cannot write at all, or write very imperfectly." (Report, p. 7.)

The Registrar-general remarks that 30 in 1000, or three per cent. of the marriageable portion of the community are married annually, the portion therefore whose signatures appear on the marriage registers of a single year is sufficiently small to be affected by accidental circumstances; and it cannot safely be asserted that the 33 in 1000, from whose signatures we would draw an inference respecting the other 970, may not happen to consist of more than the proportionate number of uneducated persons. With this caution, then, against drawing a conclusion regarding the education of any particular county or district from the returns of a single year, we are furnished with the educational result of these returns for the year ending June 30, 1839. In the metropolis, the proportion per cent. of those who, on marrying, signed with a mark was, men 12, women 24, mean 18; in the south-eastern counties, men 32, women 40, mean 36 ; in the south midland counties, men 43, women 53, mean 48 ; in the eastern counties, men 45, women 52, mean 48 ; in the south-western counties, men 31, women 47, mean 38 ; in the western counties, men 40, women 54, mean 47; in the north midland counties, men 32, women 50, mean 36 ; in the north-western counties, men 39, women 63, mean 51; in Yorkshire, men 34, women 49, mean 41; in the northern counties, men 21, women 42, mean 31 ; in Monmouthshire and Wales, men 48, women 70, mean 59.

The main points of the whole case are, that the state of education, at least among the adult population, is most deplorable for a country professedly the most civilized in the world, for that, in the whole of England and Wales, out of 121,083 couples married, there were 40,587 men and 58,959 women who could not write; that the metropolis stands decidedly superior to the rest of England in respect of education; that, next to the metropolis, the north of England (Durham, Northumberland, Cumberland, and Westmorland) is superior; that the principal deficiency is in Lancashire, Bedfordshire, Monmouthshire, and Wales; and that the men are superior to the women throughout the country in the proportion of 33 to 49.

Our high estimate of the merits of this volume is sufficiently manifest from the length to which our extracts have extended, and the remarks with which they have been interspersed. Perhaps we cannot express our opinion more decidedly than by saying that the second report has fulfilled to the utmost the promise of the first.

